# Efficient Expression of Soluble Recombinant Protein Fused with Core-Streptavidin in Bacterial Strain with T7 Expression System

**DOI:** 10.3390/mps3040082

**Published:** 2020-12-01

**Authors:** Ammar Tarar, Esmael M. Alyami, Ching-An Peng

**Affiliations:** Department of Chemical and Biological Engineering, University of Idaho, 875 Perimeter Dr, Moscow, ID 83844, USA; tara9039@vandals.uidaho.edu (A.T.); alya7030@vandals.uidaho.edu (E.M.A.)

**Keywords:** core streptavidin, fusion protein, pET-30a(+), T7-express competent *E. coli*, thymidine phosphorylase, 5′-DFUR

## Abstract

The limited amount of fusion protein transported into cytosol milieu has made it challenging to obtain a sufficient amount for further applications. To avoid the laborious and expensive task, T7 promoter-driving pET-30a(+) coding for chimeric gene of thymidine phosphorylase and core streptavidin as a model system was constructed and transformed into a variety of *E. coli* strains with T7 expression system. Our results demonstrated that the pET-30a(+)-TP-coreSA/Lemo21(DE3) system is able to provide efficient expression of soluble TP-coreSA fusion protein for purification. Moreover, the eluted TP-coreSA fusion protein tethered on biotinylated A549 carcinoma cells could effectively eliminate these malignant cells after administrating prodrug 5′-DFUR.

## 1. Introduction

The bacterium *Escherichia coli* is the most convenient and widely used system for expression of recombinant proteins [1]. The *E. coli* strains with the presence of T7 promoter-based plasmids have been extensively employed to express proteins. In this system, the T7 RNA polymerase gene is located in λ DE3 prophage of the chromosome under the control of the IPTG-inducible LacUV5 promoter [2,3]. To facilitate the immobilization of protein on biotinylated materials, expression of chimeric proteins fused with the streptavidin affinity domain has been reported using bacteria [4,5,6]. However, full length streptavidin (SA) tends to form higher order aggregates and thus has poor solubility. In contrast, core streptavidin (coreSA) is more soluble than the full-length SA. Moreover, coreSA has enhanced biotin-binding features compared to full-length SA because the removal of non-functional terminal regions promotes the binding with less steric hindrance [7]. Some chimeric proteins such as scFv and CD47 fused with coreSA affinity tag have been reported to be produced in *E. coli* [8,9,10,11]. However, a large amount of coreSA-tagged fusion proteins after expression in *E. coli* was present in an insoluble form found in inclusion bodies. These insoluble protein aggregates in *E. coli* cells could only be dissolved under strong denaturing conditions that are time-consuming and costly [12,13]. Moreover, the limited amount of fusion proteins translated into cytosol made it difficult to purify sufficient quantities necessary for further applications. Attempts have been made to express and purify coreSA-associated fusion proteins from bacterial lysate [10,11]; however, the yields were low.

In this study, we constructed a T7 promoter-based pET-30a(+) vector encoding with thymidine phosphorylase (TP)-coreSA chimeric gene as a model system for the expression of TP-coreSA fusion protein. Various bacterial strains with T7 expression system including BL21(DE3), NiCo21(DE3), Lemo21(DE3), and SHuffle^®^ competent *E. coli* were used to analyze the expression of soluble fusion protein with the goal of minimizing time- and cost-consuming steps of protein purification. Our results showed that Lemo21(DE3), transformed with the pET-30a(+) coding for TP-coreSA chimeric gene, is able to express substantial amount of soluble TP-coreSA fusion protein for time-saving and cost-effective purification. The functionality of the purified TP-coreSA fusion protein was confirmed by its effectiveness of killing biotinylated A549 lung adenocarcinoma cells tethered with TP-coreSA via biotin-SA binding and treated with prodrug 5′-DFUR.

## 2. Experimental Design

We have developed a method to construct and purify recombinant protein fused with core streptavidin. The protocols are developed step by step to guide the researchers studying custom-made coreSA-tagged fusion proteins.

### 2.1. Materials

- New England BioLabs (NEB) (Ipswich, MA, USA)
  1. High fidelity Phusion polymerase (Cat No.: M0491S)
  2. Blunt/TA Ligase Master Mix (Cat No.: M0367S)
  3. T4 DNA ligase (Cat No.: M0202S)
  4. Monarch PCR & DNA cleanup kit (Cat No.: T1030S)
  5. BL21(DE3) competent *E. coli* (Cat No.: C2527I)
  6. NiCo21(DE3) competent *E. coli* (Cat No.: C2529H)
  7. SHuffle^®^ T7 Express competent *E. coli* (Cat No.: C3029J)
  8. T7 Express Lemo21(DE3) competent *E. coli* (Cat No.: C2528J)
  9. L-rhamnose (Cat No.: B9030A)
  10. NEB^®^ 5-alpha competent *E. coli* (subcloning efficiency) (Cat No.: C2988J)
- Thermo Fisher Scientific (Waltham, MA, USA)
  1. Dulbecco’s modified Eagles’ medium (DMEM) culture media (Cat No.: 10566016)
  2. Fetal bovine serum (FBS) (Cat No.: 16000044)
  3. 0.25% trypsin-EDTA (Cat No.: 25200056)
  4. L-glutamine (Cat No.: 25030081)
  5. Biotin-X DHPE (N-((6-(biotinoyl)amino)hexanoyl)-1,2-dihexadecanoyl-sn-glycero-3-phosphoethanolamine, triethylammonium salt) (Cat No.: B1616)
  6. Streptavidin-fluorescein isothiocyanate (SA-FITC) (Cat No.: SA1001)
  7. Bacterial protein extraction reagent (B-PER) (Cat No.: 78260)
  8. Protein concentrator PES MWCO = 50 kD (Cat No.: 88541)
  9. Protease inhibitor EDTA free (Cat No.: A32961)
  10. ECL substrate (Cat No.: 35050)
  11. Tween-20 (Cat No.: 28360)
  12. Streptavidin monoclonal antibody (Cat No.: MA1-20010)
  13. Penicillin-streptomycin (Cat No.: 15140163)
  14. HisPur™ cobalt resin (Cat No.: 89964)
  15. Bicinchoninic acid (BCA) protein assay kit including bovine serum albumin (BSA) standard (Cat No.: 23227)
  16. PageRuler™ Plus prestained protein ladder, 10 to 250 kDa (Cat No.: 26620)
  17. Disposable columns, 5 mL (Cat No.:29922)
  18. Methanol (Cat No.: 18-604-352)
- Sigma-Aldrich (St. Louis, MO, USA)
  1. 5-fluorouracil (5-FU) (Cat No.: F6627)
  2. Lysogeny broth (LB media) (Cat No.: L3522)
  3. Imidazole (Cat No.: 68268)
  4. Isopropyl-β-D-thiogalactopyranoside (IPTG) (Cat No.: I5502)
  5. Potassium phosphate (Cat No.: P5655)
  6. Sodium hydroxide (Cat No.: 28-2988)
  7. Ethanol (Cat No.: 102428)
  8. Plasmid pET-30a(+) DNA (Cat No.:69909)
- Bio-Rad (Hercules, CA, USA)
  1. Tris/glycine/SDS buffer (Cat No.: 1610772EDU)
  2. Tris-buffer saline (TBS) (Cat No.: 1706435)
  3. Nitrocellulose membrane 0.2 µm (Cat No.: 1620168)
  4. Laemmli sample buffer (Cat No.: 1610737)
  5. TGX FastCast Acrylamide Solutions (SDS-PAGE gels) (Cat No.: 161-0174)
  6. 2-mercaptoethanol (Cat No.: 1610710)
- APExBIO (Houston, TX, USA)
  1. 5′-deoxy-5′-fluorouridine (5′-DFUR) (Cat No.: B5516)
- Santa Cruz Biotech (Dallas, TX, USA)
  1. Kanamycin sulfate (Cat No.: sc-257635)
  2. Dimethyl sulfoxide (DMSO) (Cat No.: sc-358801)
  3. Platelet-derived endothelial cell growth factor (PD-ECGF) monoclonal antibody (Cat No.: sc-47702)
- ATCC (Manassas, VA, USA)
  1. A549 lung adenocarcinoma cell line (Cat No.: CCL-185)
- Integrated DNA Technologies, Inc. (Coralville, IA, USA)
  1. Forward primer 5′-AGATCCGAATTCGGTGCTGCTGAAGCAGGT-3′ and reverse primer 5′-ATTATACTCGAGGGAGGCGGCGGACGGCTT-3′ for coreSA (custom-made)
  2. Forward primer 5′-GCCATGGATATCATGGCAGCCTTGATGACCCC-3′ and reverse primer 5′-GATCTCGAATTCTTGCTGCGGCGGCAGAACG-3′ for TP (custom-made)
- R&D Systems (Minneapolis, MN, USA)
  1. Mouse IgG HRP (horseradish peroxidase)-conjugated antibody (Cat. No.: HAF018)
- GenScript (Piscataway, NJ, USA)
  1. pcDNA3.1+ C-eGFP-TP plasmid (custom-made)
- QIAGEN (Germantown, MD, USA)
  1. Plasmid Miniprep kit (Cat No.: 10043)
- Research Products International Corp (Mount Prospect, IL, USA)
  1. Rapid Coomassie11 blue stain (Cat No.: RCS-50)
- Boston BioProducts (Ashland, MA, USA)
  1. Phosphate buffer saline (PBS) (Cat No.: BM-220X)
- Promega (Madison, WI, USA)
  1. Tris-Hydrochloride (Tris-HCl) (Cat No.: H5121)
- G-Biosciences (St. Louis, MO, USA)
  1. Maxi Columns (Cat. No.: 7860197)
- University of Southern California
  1. Plasmid pSTE2-215 (yol) (provided by Dr. Stanley Tahara)

### 2.2. Equipment

- SpectraMax M2e microplate reader (Molecular Devices, Sunnyvale, CA, USA)
- Chemiluminescence imager (PXi Syngene, Frederick, MD, USA)
- DMi8 microscope equipped with Leica EC3 digital color camera (Leica Microsystems, Wetzlar, Germany)
- T-100 thermocycler (Bio-Rad, Hercules, CA, USA)
- Trans-Blot^®^ semi-dry system (Bio-Rad, Hercules, CA, USA)
- C24 incubator shaker (New Brunswick Scientific, Edison, NJ, USA)
- Sorvall ST 16R centrifuge (Thermo Fisher Scientific, Waltham, MA, USA)
- Microprocessor controlled 280 series water bath (Thermo Fisher Scientific, Waltham, MA, USA)
- Mini-Protean^®^ Tetra cell and PowerPac^™^ Universal power supply (Bio-Rad, Hercules, CA, USA)

## 3. Procedure

### 3.1. Construction of TP-coreSA Encoding Plasmid

**Figure 1:**
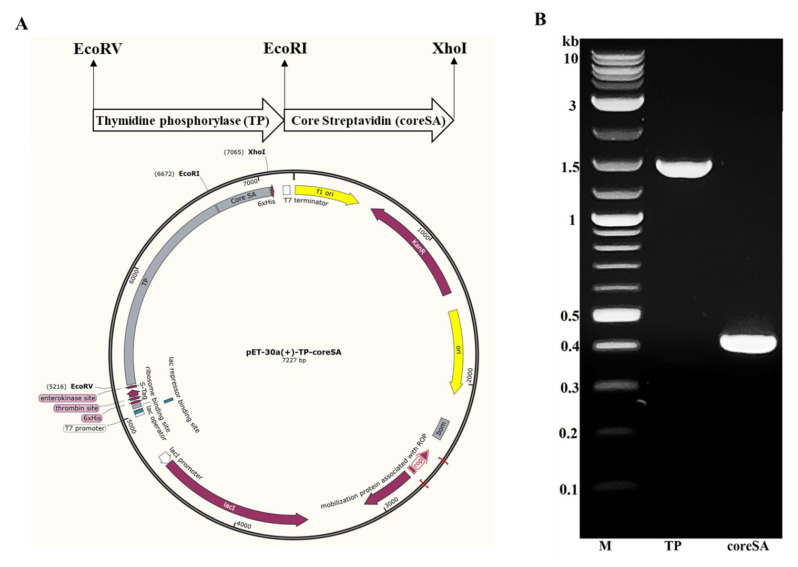
Construction of pET-30a(+)-TP-coreSA. (**A**) vector map showing the cloning site of TP and coreSA in between EcoRV (forward primer)/EcoRI (reverse primer) and EcoRI (forward primer)/XhoI (reverse primer), respectively. The direction of arrows shows the 5′ to 3′ end of each insert. (**B**) DNA agarose gel confirmation of PCR products (TP~1.4 kb, coreSA~0.4 kb) cloned from pET-30a(+)-TP-coreSA.

1. Clone TP gene sequence by polymerase chain reaction (PCR) from pcDNA3.1+ C-eGFP-TYMP using forward primer 5′-GCCATGGATATCATGGCAGCCTTGATGACCCC-3′ and reverse primer 5′-GATCTCGAATTCTTGCTGCGGCGGCAGAACG-3′. The 50 µL PCR mixture contains 1 µg of template DNA, 0.5 µM of each PCR primer, 1X Phusion HF buffer, 200 µM dNTPs, 0.5 unit of Phusion DNA polymerase, and nuclease-free water up to 50 µL. Perform PCR in T-100 thermocycler with an initial denaturation at 98 °C for 30 s, then 35 cycles of denaturation at 98 °C for 10 s, annealing at 66 °C for 30 s, and extension at 72 °C for 15 s, followed by a final extension at 72 °C for 5 min.
2. Clone core streptavidin (coreSA) gene sequence by PCR from pSTE2-215 (yol) plasmid using forward 5′-AGATCCGAATTCGGTGCTGCTGAAGCAGGT-3′ and reverse primer 5′-ATTATACTCGAGGGAGGCGGCGGACGGCTT-3′. The 50 µL PCR mixture contains 1 µg of template DNA, 0.5 µM of each PCR primer, 1X Phusion HF buffer, 200 µM dNTPs, 0.5 unit of Phusion DNA polymerase, and nuclease-free water up to 50 µL. Perform PCR in T-100 thermocycler with an initial denaturation at 98 °C for 30 s, then 35 cycles of denaturation at 98 °C for 10 s, annealing at 62 °C for 30 s, and extension at 72 °C for 15 s, followed by a final extension at 72 °C for 5 min.
3. Purify the cloned PCR product of TP and coreSA with Monarch PCR & DNA cleanup kit and analyze with 1% agarose gel electrophoresis.
4. Insert coreSA in between XhoI and EcoRI restriction sites of pET-30a(+) plasmid using T4 DNA ligase to yield pET-30a(+)-coreSA plasmid.
5. Insert TP in between EcoRI and EcoRV restriction sites of pET-30a(+)-coreSA using Blunt/TA Ligase Master Mix to yield pET-30a(+)-TP-coreSA expression vector (as shown in Figure 1).

### 3.2. Expression of TP-coreSA Encoding Plasmid

1. Transform the constructed pET-30a(+)-TP-coreSA vector into different strains of *E. coli* competent cells (i.e., BL21(DE3), NiCo21(DE3), Lemo21(DE3), and SHuffle^®^) according to manufacturer standard protocols.
2. Spread on separate agar plates supplemented with 50 µg/mL kanamycin.
3. Incubate overnight at 37 °C to screen the positive clones.
4. Pick a single colony from each agar plate and resuspend it into 5 mL LB media supplemented with 50 µg/mL kanamycin.
5. Grow overnight with shaking at 220 rpm and 37 °C to produce starter culture.
6. On the next day, dilute the starter cultures to 100 mL for SHuffle^®^, BL21(DE3), and NiCo21(DE3) separately with fresh media and keep shaking at 37 °C.
7. Induce 400 µM IPTG when optical density at 600 nm (OD_600_) of the cultures reaches 0.5, and shake for 5 h at 30 °C to induce expression.
8. Harvest the cells by centrifugation at 4500× *g* for 15 min at 4 °C.
9. Re-suspend each cell pellets in 2 mL of lysis buffer that was made by mixing B-PER with 50 mM Tris-HCl (pH 7.5) and EDTA-free protease inhibitor (1 tablet for 50 mL).
10. Wait for 10 min at room temperature and sonicate 10 times (pulse of 10 s with 10 s rest each time) with output at seven to maximize the protein extraction efficiency.
11. Finally, centrifuge lysates at 17,000× *g* for 20 min and collect the supernatant named as a soluble crude fraction and the re-suspend the pellets in 1x PBS named as an insoluble fraction.
12. To determine the optimal expression level of recombinant TP-coreSA protein with Lemo21(DE3), use 25 mL of culture media with different concentrations of L-rhamnose (0, 100, 250, 500, 750, 1000, 2000 µM) inoculated each with 0.2 mL of starter culture. Incubate all the cultures at 37 °C until OD_600_ reached 0.5.

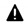
 **CRITICAL STEP:** *L-rhamnose concentration is very important and it can differ for every fusion protein of interest. So, researchers need to identify the best concentration through titer usually in the range of 0 to 2000 µM. Once the optimal concentration of L-rhamnose is identified, 100 mL of culture medium can be set up for the protein expression.*

13. Induce with 400 µM IPTG and keep shaking overnight at 22 °C with 225 rpm. Then, harvest the cells by centrifugation at 4500× *g* for 15 min, and extract the soluble crude fraction and insoluble fraction as stated above.

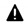
 **CRITICAL STEP:** *The IPTG concentration should remain the same, only change L-rhamnose concentration for optimal expression of fusion protein of interest.*

### 3.3. Purification of TP-coreSA Fusion Protein

1. repare binding buffer (10 mM imidazole in 1x PBS), wash buffer (10 mM imidazole in 1× PBS), and elution buffer (250 mM imidazole in 1x PBS).
2. Mix the soluble crude fraction of Lemo21(DE3) in 1:1 ratio with binding buffer and let it bind to HisPur™ cobalt resin for 1 h with gentle shaking at 4 °C.
3. Load into the column and collect the flow-through.
4. Wash with 5 resin bed volume of wash buffer and elute with 3 resin bed volume of elution buffer. (For instance, 2 mL of resin was used in our experiment, it was washed with 10 mL with wash buffer and eluted with 6 mL of elution buffer).
5. Measure OD_280_ of each collected fraction with SpectraMax M2e microplate reader to plot the elution profile.

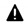
 **CRITICAL STEP:** *Always place lysates, crude proteins, insoluble fraction, and purified protein on ice during the experiments.*

**OPTIONAL** **STEP:***Concentrate the eluted protein with Protein Concentrators PES (MWCO = 50 kD) if needed. Based on the size of purified protein of interest, different MWCO might be used.*

6. For the control group study using coreSA protein only, Lemo21(DE3) is transformed with the constructed pET-30a(+)-coreSA plasmid, expressed, and purified by the aforementioned methods.

### 3.4. Characterization of TP-coreSA Fusion Protein

#### 3.4.1. BCA Protein Assay

1. Dilute the 2000 µg/mL of bovine serum albumin (BSA) stock to seven different concentrations (1500, 1000, 750, 500, 250, 125, 25 µg/mL) to prepare standards.
2. Prepare the working reagent by mixing 50:1 ratio of BCA kit reagent A with BCA kit reagent B.
3. Mix 200 µL of working reagent and 30 µL of each BSA standard (prepared in the 1st step) and elution protein (obtained from purification).
4. Incubate all samples at 37 °C for 30 min and measure the absorbance at 562 nm with SpectraMax M2e microplate reader.
5. Do all the experiments in triplicate to calculate the mean values with standard deviation.

#### 3.4.2. SDS-PAGE Analysis

1. Characterize the TP-coreSA fusion protein along with soluble crude fraction with SDS-PAGE.
2. Mix each sample with an equal amount of 2x Laemmli sample buffer containing 1% 2-mercaptoethanol and heat up to 80 °C for 10 min and briefly spin it down.
3. Load 10 µL of protein ladder and 15 µL of each sample in 12% polyacrylamide gel using 1× Tris/glycine/SDS buffer and run for 40 min at 200 volts.
4. Use the Rapid Coomassie11 blue stain to stain the gel and de-stain overnight with slow de-stained solution (7.5% methanol, 5% acetic acid).

#### 3.4.3. Western Blot Analysis

1. For Western blot, again perform the SDS-PAGE according to the aforementioned protocols, but instead of staining the gel, transfer to nitrocellulose membrane in Trans-Blot^®^ semi-dry system.
2. Block the membrane for 1 h using blocking buffer (5% BSA in TBST (Tris-buffered saline with 0.1% Tween-20)) at room temperature with constant gentle shaking.
3. Wash 3 times with TBST buffer, then allow the membrane to react with streptavidin monoclonal antibody solution overnight at 4 °C.

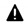
 **CRITICAL STEP:** *Prepare the working solution of primary antibody by 1:1000 dilution of antibody stock in blocking buffer. The dilution factor can be changed and titered according to the results. The reaction time can be decreased to 1 h if shaking at room temperature.*

4. For the second Western blot, redo the 1st and 2nd step and use anti-TP (PD-ECGF) monoclonal antibody for the 3rd step.
5. Wash the membranes again 3 times to remove any excess of primary antibodies and incubate for 1 h at room temperature in secondary antibody solution (1:100 dilution of mouse IgG HRP-conjugated antibody in blocking buffer).

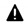
 **CRITICAL STEP:** *Although both streptavidin and TP (PD-ECGF) Western blots are recognized by the same secondary antibody, incubate the membranes separately in secondary antibody solution by preparing two working solution of secondary antibody.*

6. Finally, wash the membranes again and detect the fusion protein with horseradish peroxidase activity using ECL substrate by taking imaging through chemiluminescence imager.

#### 3.4.4. TP-coreSA Enzyme Activity and Kinetics

1. Mix 20 µL of eluted TP-coreSA protein in ice-cold 40 µL reaction mixture containing 10 mM 5′-DFUR and 25 mM potassium phosphate buffer (pH = 7.4).
2. Measure the initial absorbance as a baseline at 305 nm with SpectraMax M2e microplate reader.
3. Incubate the reaction mixture at 37 °C for 2 h and terminate the reaction by the addition of 500 mM sodium hydroxide.
4. Measure the final absorbance at 305 nm, and then calculate the amount 5-fluorouracil (5-FU) produced using the calibration curve of 5-FU at 305 nm [14]. TP enzyme activity is expressed as the amount of 5-FU (µmole) produced per purified fusion protein (µg) per hour (µmole/µg/h).
5. For enzyme kinetics, repeat all steps (from 1 to 4) with various concentrations of 5′-DFUR to react with TP-coreSA fusion protein for 2 h and obtain enzyme activities.
6. Plot enzyme activities versus corresponding concentrations of 5′-DFUR.
7. Use the Michaelis-Menten equation to do a curve fitting of the data points.

#### 3.4.5. Surface Biotinylation of Cells

1. Inoculate A549 cells in 6-well plates containing DMEM supplemented with 10% FBS and 1% penicillin-streptomycin, and cultivate in a 5% CO_2_ incubator balanced with humidifier air.
2. Replace the culture media after 24 h with fresh media supplemented with 0.02 mg/mL Biotin-X DHPE and incubate for further 48 h.
3. Confirm the surface biotinylation by incubating in SA-FITC for 1 h and let streptavidin bind with the biotinylated surface of cells.
4. Gently wash the cells again with 1x PBS to discard any unbounded SA-FITC, and take the images with a Leica DMi8 microscope equipped with Leica EC3 digital color camera.

### 3.5. Cytotoxicity Studies

1. Add 100 μL of TP-coreSA elution protein and incubate for 1 h to let it bind to the biotinylated A549 cells.
2. Add 100 µM of 5′-DFUR into each well and incubate for 4 days.
3. Culture positive and negative control experiment at the same time.
4. Calculate the cell viability at the end of the experiment using trypan blue staining and hemocytometer.

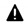
 **CRITICAL STEP:** *Carry out the experiments in triplicate and calculate the percentage of cancer cell viability compared to the control groups.*

## 4. Results and Discussion

### 4.1. Expression of pET-30a(+)-TP-coreSA

**Figure 2:**
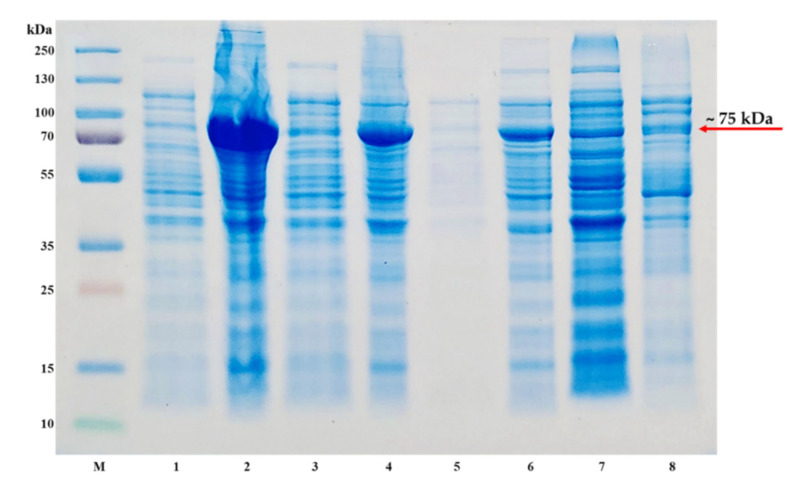
Expression of TP-coreSA fusion protein using different strains of competent cells. SDS-PAGE is imaged as (**M**) protein ladder, (**1**) NiCo21(DE3) soluble fraction, (**2**) NiCo21(DE3) pellets, (**3**) BL21(DE3) soluble fraction, (**4**) BL21(DE3) pellets, (**5**) SHuffle^®^ soluble fraction, (**6**) SHuffle^®^ pellets, (**7**) Lemo21(DE3) soluble fraction, (**8**) Lemo21(DE3) pellets.

The pET-30a(+)-TP-coreSA vector was constructed by inserting gene sequences of core streptavidin (coreSA) and thymidine phosphorylase (TP) in between EcoRI/XhoI and EcoRV/EcoRI respectively, and verified using agarose gel as shown in Figure 1. Furthermore, the cloned sequence of TP and coreSA in pET-30a(+)-TP-coreSA was confirmed by the Sanger sequencing performed by GenScript. The molecular weight of fusion protein is estimated to be 75 kDa as the TP being 1458 bp (~55 kDa), coreSA being 387 bp (~15 kDa), and others (S tag, thrombin site, 6xHis, enterokinase site) being 135 bp (~5 kDa). The pET-30a(+)-TP-coreSA vector was then transformed into different strains of competent cells—Lemo21(DE3), Shuffle^®^, BL21(DE3), and NiCo21(DE3)—to check the expression of the fusion protein in soluble and insoluble fraction. As shown in Figure 2, the SDS-PAGE has a very strong expression around 75 kDa in the insoluble fraction of NiCo21(DE3) (lane 2) as compared to a very light band in the soluble fraction (lane 1). The amount of insoluble fusion protein expressed in BL21(DE3) was much less than the one shown in lane 2; however, its soluble fraction quantity was slightly higher than one obtained by NiCo21(DE3) (see lane 1). For SHuffle^®^ expression cells, the level of TP-coreSA fusion protein in an insoluble form (lane 6) was lower than the one from BL21(DE3) (see lane 6). Additionally, there was nominal no detection of fusion protein in soluble fraction (lane 5). Taken altogether, large amount of TP-coreSA fusion protein was presented in inclusion bodies (i.e., cell pellets) when using NiCo21(DE3), BL21(DE3), and SHuffle^®^ bacterial strains. However, the expression with Lemo21(DE3) led to more soluble fraction (lane 7) as compared to its insoluble counterpart (lane 8).

**Figure 3:**
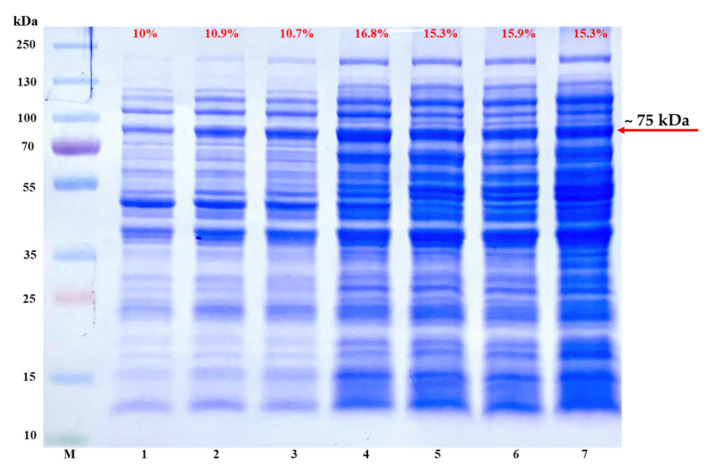
The effect of L-rhamnose concentration on recombinant protein expression in Lemo21(DE3) competent cells transformed with pET-30a(+)-TP-coreSA vector. The SDS-PAGE is imaged as (**M**) protein ladder, (**1**) control (without L-rhamnose), (**2**–**7**) starting from lane 2 with 100 µM concentration of L-rhamnose followed by 250, 500, 750, 1000, and 2000 µM, respectively. The percentages of TP-coreSA in crude protein for all lanes were calculated and added into the gel images showing on top of each lane (in red).

Generally, T7 expression of proteins targeted to the Sec translocase often leads to accumulation of inclusion bodies, hence making the protein of interest in an insoluble form [12]. According to the results shown in Figure 2, the issue of TP-coreSA fusion protein prone to insoluble expression can be alleviated by using Lemo21(DE3), which is a variant strain of BL21(DE3) that offers tunable T7 expression by controlling the level of T7 lysozyme which is a natural inhibitor of T7 RNA polymerase [15]. The level of T7 lysozyme is modulated by varying L-rhamnose concentration in expression culture. A variety of concentrations (0, 100, 250, 500, 750, 1000, 2000 µM) of L-rhamnose were titrated to optimize the expression level of soluble fractions. As illustrated in Figure 3, L-rhamnose concentration ≥ 500 µM was able to finely tuned high level of TP-coreSA fusion protein expressed in soluble fractions. For each lane in Figure 3, the amounts of soluble crude protein and its corresponding eluted TP-coreSA fusion protein were determined by BCA assay. The percentages of TP-coreSA fusion protein in soluble crude protein were calculated to be 10%, 10.9%, 10.7%, 16.8%, 15.3%, 15.9%, and 15.3% for 0, 100, 250, 500, 750, 1000, and 2000 µM of L-rhamnose treatment, respectively. It is noteworthy that the eluted fusion protein from 500 µM or higher of L-rhamnose soluble fraction was about 3-fold higher than the one without L-rhamnose, although the background was also expressed more for other proteins (Figure 3 lane 4–7).

### 4.2. Purification of TP-coreSA Fusion Protein

**Figure 4:**
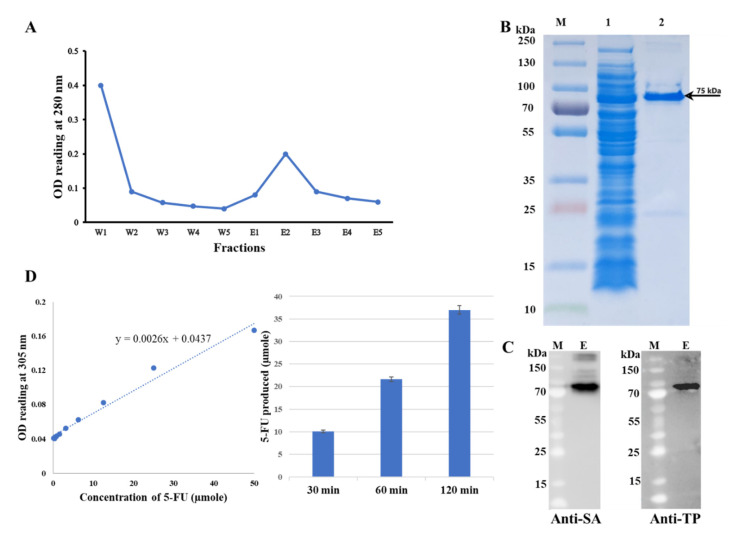
(**A**) Elution profile of TP-coreSA fusion protein using five washes and five elutions; (**B**) SDS-PAGE imaged as (M) protein ladder, (1) crude bacterial lysate, (2) purified TP-coreSA fusion protein; (**C**) Western blot against anti-TP and anti-SA, (M) is protein ladder, (E) is purified TP-coreSA; (**D**) Measurement of enzyme activity of TP-coreSA fusion protein using a microplate reader. The change in absorbance at 305 nm is used to calculate the 5-FU released after incubation of TP-coreSA in the presence of 5′-DFUR for up to 2 h with the help of 5-FU calibration curve.

The crude protein was extracted using Lemo21(DE3) competent cells with 500 µM of L-rhamnose as explained in the previous section. The TP-coreSA fusion protein was purified from crude fraction by affinity chromatography using a disposable column packed with cobalt resin. Although the yield of the fusion protein is lower using cobalt resin instead of nickel resin, it will help to reduce the nonspecific binding and results in more purified protein. Moreover, it also eases the purification process as it does not need to optimize the imidazole concentration in binding and washing buffer, which is usually needed while using nickel resin. The elution profile of the purification system shows the peak OD_280_ at elution 2 as given in Figure 4A.

### 4.3. Characterization of TP-coreSA Fusion Protein

#### SDS-PAGE Analysis, Western Blot, TP-coreSA Enzyme Activity and Kinetics

The purified TP-coreSA fusion protein was concentrated five to ten folds by a protein concentrator PES (MWCO = 50 kD). As shown in Figure 4B, SDS-PAGE displayed the purified single band of TP-coreSA fusion protein (75 kDa) along with soluble crude fraction. It was further characterized through Western blot; the purified fusion protein was detected against anti-TP and anti-SA monoclonal antibodies confirming the fusion protein at 75 kDa location (Figure 4C). The concentration of fusion protein was calculated as 217 ± 6.5 µg/mL using the BCA assay. SpectraMax M2e microplate reader was used to calculate the enzyme activity of TP-coreSA fusion protein by incubating with 5′-DFUR as described in the Section 3.4.4. As shown in Figure 4D, the change in absorbance at 305 nm is used to calculate the 5-FU produced from the conversion of 5′-DFUR by TP-coreSA enzyme using 5-FU calibration curve at 305 nm. The obtained absorbance changes after 30, 60, and 120 min were used to calculate 5-FU produced as 10.1 ± 0.33, 21.6 ± 0.52, and 37 ± 0.98 µmole, respectively. Since 20 µL of purified fusion protein was used, the TP-coreSA enzyme activity was calculated to be 4.98 ± 0.13 µmole/µg/h. It should be noted that the enzyme activity obtained here is much higher than the reported values ranged from 0.269 to 3.383 µmole/mg/h [14,16,17,18]. This is because the literature-reported values of TP enzyme activity were obtained by dividing total amount of crude protein; however, in this study, the enzyme activity of TP-coreSA was calculated by dividing the purified protein, hence, it is much higher than the values reported. For the study of enzyme kinetics, enzyme activities (µmole/µg/h) plotted with their corresponding concentrations of substrate (i.e., 5′-DFUR) were curve fitted well with the Michaelis-Menten equation (data not shown).

### 4.4. Surface Biotinylation of A549 Cells

**Figure 5:**
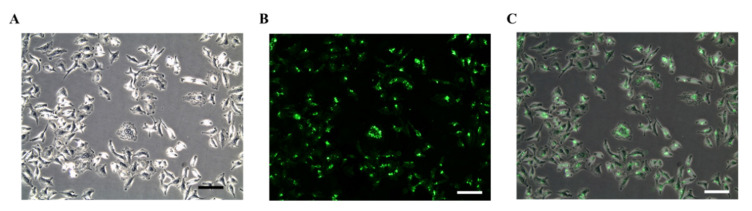
Phase contrast (**A**), fluorescence (**B**), and overlay images (**C**) of A549 cells treated with 0.02 mg/mL biotin-X DHPE followed by the treatment of SA-FITC (scale bar donates 100 µm).

The biotinylated A549 cells were incubated with SA-FITC for 1 h to allow streptavidin-biotin binding. After that, images were taken under a fluorescent microscope. Figure 5A shows phase contrast grayscale image of biotinylated A549 cells, and Figure 5B is the fluorescent image of the same location showing green illuminance of bound SA-FITC. The overlay image (normal grayscale plus fluorescent) (Figure 5C) demonstrates that the approach used for cell biotinylation did not affect cell viability and its morphology.

### 4.5. Cytotoxicity Studies

**Figure 6:**
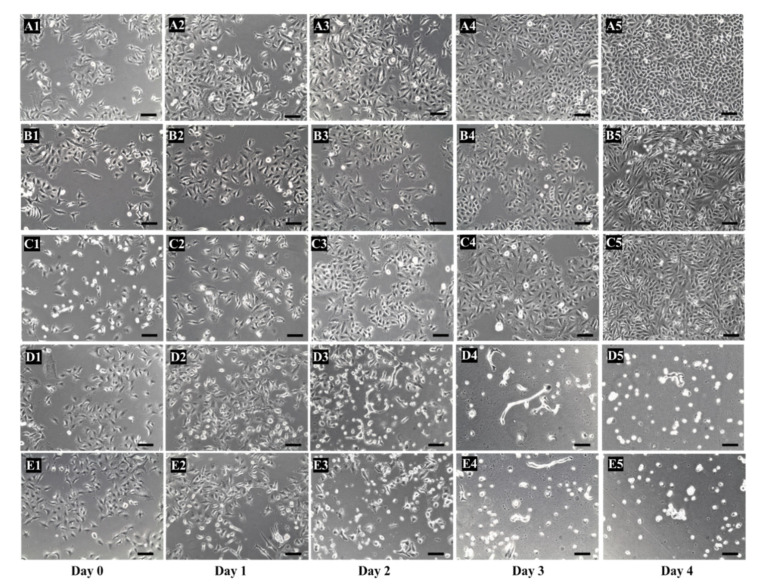
Photomicrographic images of A549 cell over the course of 4-day cultivation. (**A1**–**A5**) negative control group 1: untreated A549 cells; (**B1**–**B5**) negative control group 2: A549 cells treated with 5′-DFUR; (**C1**–**C5**) negative control group 3: A549 cells tethered with coreSA protein and treated with 5′-DFUR; (**D1**–**D5**) A549 cells decorated with TP-coreSA fusion protein and treated with 100 µM of 5′-DFUR; (**E1**–**E5**) positive control group—A549 cells treated with 100 µM of 5-FU (scale bar donates 100 µm).

Several studies have shown the anti-cancer effectiveness of TP as it converts prodrug 5′-DFUR to cytotoxic chemotherapy drug 5-FU [19,20,21]. The functionality of purified TP-coreSA fusion protein was examined via its anti-cancer activity after treating malignant cells in the presence of prodrug 5′-DFUR. The A549 cancer cells were cultured up to 40% confluence, biotinylated, and tethered with TP-coreSA fusion protein. After administrated with 100 µM of 5′-DFUR, A549 cells were eradicated in four days (see Figure 6D). This indicated that 5′-DFUR (prodrug) was enzymatically catalyzed into 5-FU with the aid of TP-coreSA fusion protein anchored on the surface of A549 cells via biotin-SA binding. To confirm the anticancer effectiveness is attributed to the formation of toxic 5-FU from prodrug 5′-DFUR, three control experiments were conducted as following: (i) biotinylated A549 cells without any treatment, (ii) biotinylated A549 cells treated with 5′-DFUR, and (iii) biotinylated A549 cells tethered with coreSA protein and treated with 5′-DFUR. As shown in Figure 6A–C, cells in these three control groups grew continuously and reached 100% confluency in 4 days. This means the endogenous expression of TP (if there is any) in A549 cells was not enough to convert the 5′-DFUR to 5-FU to kill cells. Moreover, the positive control group (Figure 6E) was separately carried out by direct administration of 100 µM of 5-FU and revealed similar cell morphological change and cell death pattern illustrated in Figure 6D. However, the elimination of cells in the positive control was slightly quick with most of the cells killed at day 2 and 3 (Figure 6, E3–E4) as compared to 5′-DFUR that showed the eradication occurred at day 3 and 4 (Figure 6, D3–D4). It is speculated that the conversion yield of 5′-DFUR into 5-FU was not 100%; hence, this inefficient conversion led to a delay in killing cancer cells [22].

**Figure 7:**
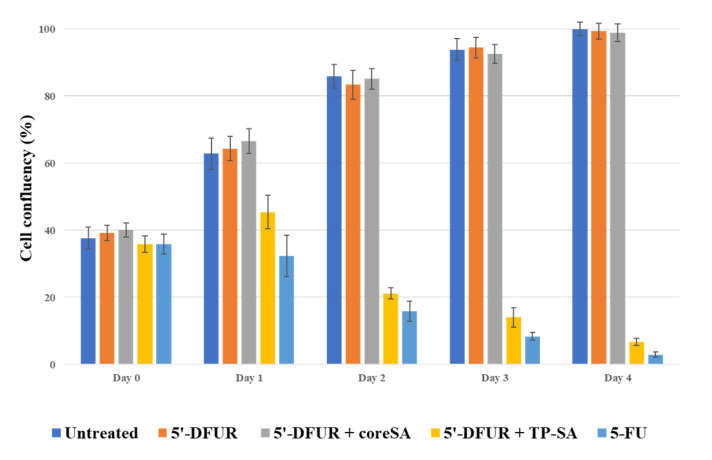
Viable cell confluency of A549 cells after treatment with nothing, 5′-DFUR, 5′-DFUR + coreSA, 5′-DFUR + TP-coreSA, and 5-FU for 4 days of culture. Cell inoculation for all groups was about 38% confluency.

Figure 7 showed the cell confluency on a daily basis. For all negative control groups, A549 cells with 38% confluency at the inoculation continued to grow and reached 100% confluency in four days. Conversely, the prodrug treated with TP-decorated cells showed little increase in confluency on day 1 and then started dying and ended up with < 6% of viable cells on day 4, while cells treated directly with 5-FU showed a decrease in cell viability on the very next day after cytotoxic drug administration and had about 3% cell confluency on day 4.

## 5. Conclusions

In this study, pET-30a(+) plasmid encoding with TP-coreSA fusion gene was constructed. The pET-30a(+)-TP-coreSA plasmid was transformed and expressed in a variety of *E. coli* strains with T7 expression system. Our results demonstrated that Lemo21(DE3) produced substantial amount of TP-coreSA fusion protein in soluble fraction which was then purified by affinity chromatography. The optimized pET-30a(+)-TP-coreSA/Lemo21(DE3) system is time-saving and cost-effective because it avoids denaturing and refolding processes involved with protein expressed in inclusion bodies. The purified TP-coreSA fusion protein was characterized by enzyme activity assay. The anticancer effectiveness of TP-coreSA fusion protein was revealed by tethering it on biotinylated A549 cancer cells and treated with prodrug 5′-DFUR.

## References

[1] Rosano G.L., Ceccarelli E.A. (2014). Recombinant protein expression in Escherichia coli: Advances and challenges. Front. Microbiol..

[2] Arditti R., Scaife J.G., Beckwith J.R. (1968). The nature of mutants in the lac promoter region. J. Mol. Biol..

[3] Studier F.W., Rosenberg A.H., Dunn J.J., Dubendorff J.W. (1990). Use of T7 RNA polymerase to direct expression of cloned genes. Heterotrimeric G-Protein Eff..

[4] Walsh M.K., Swaisgood H.E. (1994). AnEscherichia coli plasmid vector system for production of streptavidin fusion proteins: Expression and bioselective adsorption of streptavidin-?-galactosidase. Biotechnol. Bioeng..

[5] Lee P., Swaisgood H. (1998). Cloning and expression of a streptavidin-lipase fusion gene in Escherichia coli and characterization of the immobilized fusion protein. Enzym. Microb. Technol..

[6] Goshorn S., Sanderson J., Axworthy D., Lin Y., Hylarides M., Schultz J. (2001). Preclinical Evaluation of a Humanized NR-LU-10 Antibody-Streptavidin Fusion Protein for Pretargeted Cancer Therapy. Cancer Biother. Radiopharm..

[7] Pandori M.W., Sano T., Chen X., Smith C.L., Cantor C.R. (1995). Recombinant Core Streptavidins: A Minimum-Sized Core Streptavidin Has Enhanced Structural Stability and Higher Accessibility to Biotinylated Macromolecules. J. Biol. Chem..

[8] Dübel S., Breitling F., Kontermann R., Schmidt T., Skerra A., Little M. (1995). Bifunctional and multimeric complexes of streptavidin fused to single chain antibodies (scFv). J. Immunol. Methods.

[9] Wang W.W.-S., Das D., McQuarrie S.A., Suresh M.R. (2007). Design of a bifunctional fusion protein for ovarian cancer drug delivery: Single-chain anti-CA125 core-streptavidin fusion protein. Eur. J. Pharm. Biopharm..

[10] Hsu Y.-C., Acuña M., Tahara S.M., Peng C.-A. (2003). Reduced phagocytosis of colloidal carriers using soluble CD47. Pharm. Res..

[11] Salehi N., Peng C.-A. (2016). Purification of CD47-streptavidin fusion protein from bacterial lysate using biotin-agarose affinity chromatography. Biotechnol. Prog..

[12] Singh A., Upadhyay V., Upadhyay A.K., Singh S.M., Panda A.K. (2015). Protein recovery from inclusion bodies of Escherichia coli using mild solubilization process. Microb. Cell Factories.

[13] Drew D., Fröderberg L., Baars L., De Gier J.W. (2003). Assembly and overexpression of membrane proteins in Escherichia coli. Biochim. Biophys. Acta (BBA) Biomembr..

[14] Patterson A., Zhang H., Moghaddam A., Bicknell R., Talbot D., Stratford I.J., Harris A.L., Patterson A.V. (1995). Increased sensitivity to the prodrug 5′-deoxy-5-fluorouridine and modulation of 5-fluoro-2′-deoxyuridine sensitivity in MCF-7 cells transfected with thymidine phosphorylase. Br. J. Cancer.

[15] Wagner S., Klepsch M.M., Schlegel S., Appel A., Draheim R., Tarry M., Högbom M., Van Wijk K.J., Slotboom D.J., Persson J.O. (2008). Tuning Escherichia coli for membrane protein overexpression. In Proceedings of the Proceedings of the National Academy of Sciences. Proc. Natl. Acad. Sci. USA.

[16] Sawdon A.J., Zhang J., Wang X., Peng C.-A. (2018). Enhanced Anticancer Activity of 5′-DFUR-PCL-MPEG Polymeric Prodrug Micelles Encapsulating Chemotherapeutic Drugs. Nanomaterials.

[17] Miyadera K., Sumizawa T., Haraguchi M., Yoshida H., Konstanty W., Yamada Y., Akiyama S.-I. (1995). Role of thymidine phosphorylase activity in the angiogenic effect of platelet derived endothelial cell growth factor/thymidine phosphorylase. Cancer Res..

[18] Evrard A., Cuq P., Ciccolini J., Vian L., Cano J.-P. (1999). Increased cytotoxicity and bystander effect of 5-fluorouracil and 5′-deoxy-5-fluorouridine in human colorectal cancer cells transfected with thymidine phosphorylase. Br. J. Cancer.

[19] López-Estévez S., Ferrer G., Torres-Torronteras J., Mansilla M.J., Casacuberta-Serra S., Martorell L., Hirano M., Martí R., Barquinero J. (2014). Thymidine phosphorylase is both a therapeutic and a suicide gene in a murine model of mitochondrial neurogastrointestinal encephalomyopathy. Gene Ther..

[20] Lettieri R., D’Abramo M., Stella L., La Bella A., Leonelli F., Giansanti L., Venanzi M., Gatto E. (2018). Fluorescence and computational studies of thymidine phosphorylase affinity toward lipidated 5-FU derivatives. Spectrochim. Acta Part A Mol. Biomol. Spectrosc..

[21] Tsuneyoshi K., Haraguchi M., Hongye Z., Gotanda T., Tachiwada T., Sumizawa T., Furukawa T., Baba M., Akiyama S.-I., Nakagawa M. (2006). Induction of thymidine phosphorylase expression by AZT contributes to enhancement of 5′-DFUR cytotoxicity. Cancer Lett..

[22] Ishikawa T., Utoh M., Sawada N., Nishida M., Fukase Y., Sekiguchi F., Ishitsuka H. (1998). Tumor selective delivery of 5-fluorouracil by capecitabine, a new oral fluoropyrimidine carbamate, in human cancer xenografts. Biochem. Pharmacol..

